# Survey of Endosymbionts in the *Diaphorina citri* Metagenome and Assembly of a *Wolbachia* wDi Draft Genome

**DOI:** 10.1371/journal.pone.0050067

**Published:** 2012-11-16

**Authors:** Surya Saha, Wayne B. Hunter, Justin Reese, J. Kent Morgan, Mizuri Marutani-Hert, Hong Huang, Magdalen Lindeberg

**Affiliations:** 1 Department of Plant Pathology and Plant-Microbe Biology, Cornell University, Ithaca, New York, United States of America; 2 USDA-ARS, U.S. Horticultural Research Laboratory, Fort Pierce, Florida, United States of America; 3 Genformatic, LLC., Alpharetta, Georgia, United States of America; 4 School of Information, University of South Florida, Tampa, Florida, United States of America; Technion-Israel Institute of Technology Haifa 32000 Israel., Israel

## Abstract

*Diaphorina citri* (Hemiptera: Psyllidae), the Asian citrus psyllid, is the insect vector of *Ca*. Liberibacter asiaticus, the causal agent of citrus greening disease. Sequencing of the *D. citri* metagenome has been initiated to gain better understanding of the biology of this organism and the potential roles of its bacterial endosymbionts. To corroborate candidate endosymbionts previously identified by rDNA amplification, raw reads from the *D. citri* metagenome sequence were mapped to reference genome sequences. Results of the read mapping provided the most support for *Wolbachia* and an enteric bacterium most similar to *Salmonella*. *Wolbachia*-derived reads were extracted using the complete genome sequences for four *Wolbachia* strains. Reads were assembled into a draft genome sequence, and the annotation assessed for the presence of features potentially involved in host interaction. Genome alignment with the complete sequences reveals membership of *Wolbachia* wDi in supergroup B, further supported by phylogenetic analysis of FtsZ. FtsZ and Wsp phylogenies additionally indicate that the *Wolbachia* strain in the Florida *D. citri* isolate falls into a sub-clade of supergroup B, distinct from *Wolbachia* present in Chinese *D. citri* isolates, supporting the hypothesis that the *D. citri* introduced into Florida did not originate from China.

## Introduction

The biology and ecology of *Diaphorina citri*, the Asian citrus psyllid, has attracted significant attention given its role as vector of *Ca.* Liberibacter asiaticus, causal agent of citrus greening disease (huanglongbing). *D. citri* has wide geographic distribution, likely originating in Asia and spreading through the Western Hemisphere in recent decades [1]. Though less well characterized than other members of the Sternorrhyncha including aphids, coccids and whiteflies, the role of *D. citri* as a vector of *Ca.* L. asiaticus has prompted initiation of *D. citri* genome sequencing for improved characterization of psyllid biology. One of the chief motivations for sequencing the psyllid and its community of bacterial endosymbionts is to gain insight into the potential contributions of the endosymbiont population to the fitness of the insect and to transmission of *Ca.* L. asiaticus. Endosymbionts have been shown to significantly impact diverse processes in host insects including nutritional status [2], reproduction [3], lifespan [4], and resistance to insecticides [5]. Sequence data on the endosymbionts can additionally provide valuable data for elucidating population dynamics.

Psyllids are host to a variety of bacterial endosymbionts including the obligate endosymbiont gamma-proteobacterial *Ca.* Carsonella, present in specialized bacteriocytes within the insect. Genome sequences for *Ca.* Carsonella strains from multiple psyllid genera have been determined, revealing them to be the most highly reduced bacterial genomes characterized to date [6], [7]. A second endosymbiont, *Wolbachia*, is present in psyllids and a wide variety of other insects [8] and has been found in various tissues including bacteriocytes [9] and other somatic and reproductive tissues [10]. Characterization of Wolbachia is of particular interest given the extent of its impact on host biology and the potential for controlling disease-vectoring insects like *D. citri* by manipulation of their resident Wolbachia strains. The presence of *Ca.* Carsonella and *Wolbachia* has been confirmed in *D. citri* isolates from different geographic origins [11]–[13].

Microbial surveys of whole *D. citri* isolates from Indonesia and Florida, conducted by PCR-amplification of ribosomal RNA, point to the presence of diverse additional bacteria. Subandiyah et al identified a beta-proteobacterium most closely related to the genera *Oxalobacter/Herbaspirillium* and an enteric bacteria similar to *Arsenophonus* in *D. citri* isolates from Indonesia [11]. Amplification of eubacterial rDNA from *D. citri* collected in Florida revealed the presence of eight bacteria in addition to *Ca.* Carsonella and *Wolbachia* [12]. Similar surveys conducted on the potato psyllid, *Bactericera cockerelli*, vector of another *Ca.* Liberibacter pathogen, indicate the presence of *Ralstonia*, *Bradyrhizobium*, and *Staphylococcus* [14], and in the second study, the presence of *Acinetobacter* and *Methylibium* [15].

**Figure 1:**
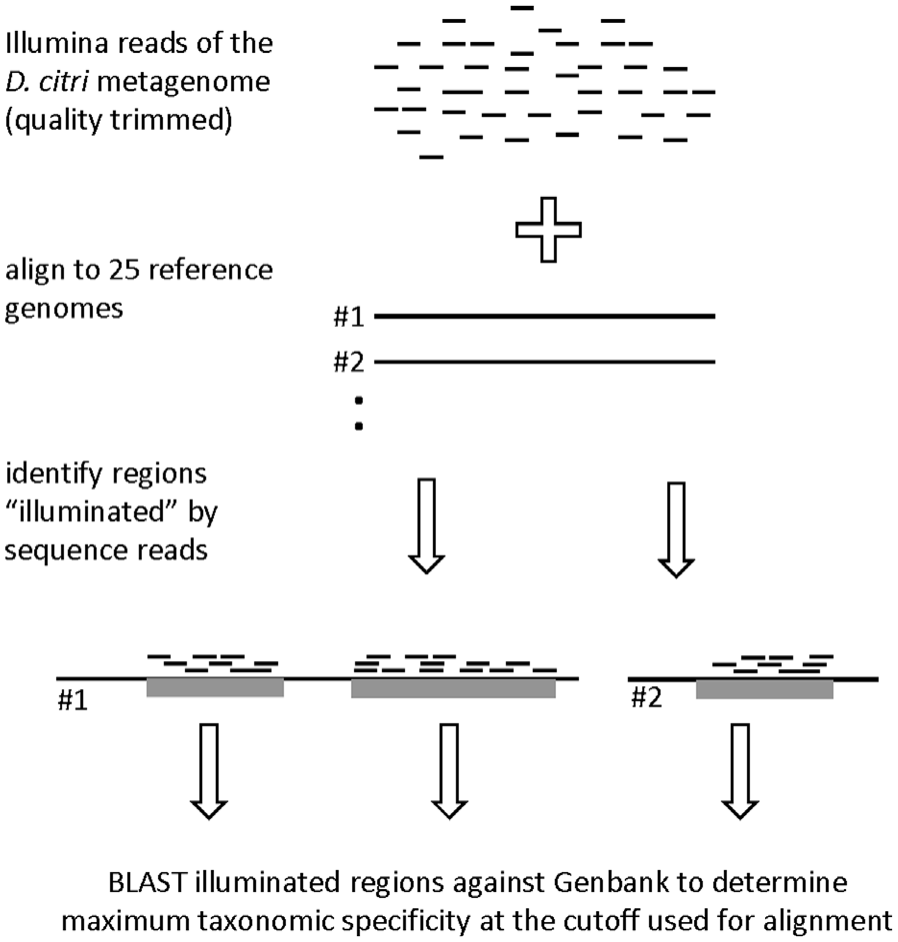
Workflow used to identify regions of reference genomes illuminated by sequence reads from the *Diaphorina citri* metagenome data set.

To facilitate more detailed characterization of psyllid biology along with genome-based characterization of endosymbionts, genome and transcriptome datasets for the *D. citri* metagenome have been generated by the International Psyllid Genome Consortium, with sequencing efforts led by the USDA-ARS Subtropical Insects Research Unit (Ft. Pierce, FL). The *D. citri* metagenome is composed of sequence reads from the psyllid in addition to those of component endosymbionts, with the extent of coverage varying in relation to the relative titer of the bacteria and the properties of the sequences themselves. A major goal of the present study is to determine the extent to which the sequence reads from the *D. citri* metagenome confirm the endosymbiont diversity previously identified by rDNA amplification. A second goal of this study is the characterization of the *Wolbachia* endosymbiont (wDi) given that preliminary analyses suggest sufficient coverage for generation of a draft genome sequence. Enhanced understanding of *Wolbachia* wDi biology is a priority given that high titers are correlated to *Ca.* L. asiaticus transmission [16] and in other systems the manipulation of *Wolbachia* has proven an effective strategy for reducing disease transmission [13]. wDi genome data will additionally provide an inventory of candidate host-interaction factors as well as providing insight into the phylogenetic placement of wDi among *Wolbachia* isolates worldwide.

**Table 1:** Endosymbionts identified in *D. citri* and *B. cockerelli* isolates by rDNA sequencing and evidence for sequences derived from these candidates in the *D. citri* metagenome.

*D. citri*-FL^a^	*D.* *citri*-SEA	*B. cockerelli*	*B. cockerelli*	Template genome ID	Template genome name	Template genome size	Cumulative size illuminated^b^	% of template genome
x	x	x	x	NC_010981	*Wolbachia* endosymbiont of *Culex quinquefasciatus*	1,482,455	1,208,733	81.5
x	x		x	NC_008512	*Candidatus* Carsonella ruddii PV	159,662	5,318	3.3
gen. indet.				NC_004631	*Salmonella enterica* subsp. *enterica* Typhi Ty2	4,791,961	604,715	12.6
gen. indet.				NC_013850	*Klebsiella variicola* At-22	5,458,505	387,559	7.1
sp. indet.		sp. indet.		NC_013893	*Staphylococcus lugdunensis* HKU09-01	2,658,366	0	0
sp. indet.		sp. indet.		NC_004461	*Staphylococcus epidermidis* ATCC 12228	2,499,279	0	0
sp. indet.		sp. indet.		NC_014925	*Staphylococcus pseudintermedius* HKU10-03	2,617,381	0	0
sp. indet.		sp. indet.		NC_010079	*Staphylococcus aureus* subsp. *aureus* USA300	2,872,915	0	0
x				NC_015138	*Acidovorax avenae* subsp. avenae ATCC 19860	5,482,170	16,249	3
sp. indet.			sp. indet.	NC_014259	*Acinetobacter* sp. DR1 chromosome	4,152,543	3,418	<1
sp. indet.			sp. indet.	NC_010611	*Acinetobacter baumannii* ACICU chromosome	3,904,116	1,842	<1
x				NC_009659	*Janthinobacterium* sp. Marseille	4,110,251	742	<1
x	x			NC_014323	*Herbaspirillum seropedicae* SmR1 chromosome	5,513,887	3,927	<1
			x	NC_008825	*Methylibium petroleiphilum* PM1 chromosome	4,044,195	3,608	<1
		x		NC_014311, NC_014310	*Ralstonia*	5605618	2,732	<1
		x		NC_004463	*Bradyrhizobium*		0	0
				NC_012985	*Candidatus* Liberibacter asiaticus str. Psy62	1,226,704	0	0

a Gen. indet. and sp. indet. indicate genera or species listed in adjacent rows that could not be distinguished by rDNA sequencing.

b Regions in the template reference genome to which unassembled sequence reads from the D. citri metagenome mapped.

## Methods

### Psyllid Maintenance, DNA Preparation, and DNA Sequencing

Psyllids were maintained on citrus and orange jasmine at the USDA-ARS laboratory in Fort Pierce, FL. Adult and 5th instar psyllids of mixed genders were collected and stored at −80°C in 1.5 ml microcentrifuge tubes. Frozen samples were thawed and resuspended with β-mercaptoethanol in 500 µl RLT buffer (Qiagen, Valencia, CA). Bacterial DNA was lysed with 0.1-mm glass beads in Qiagen Tissue Lyser (Qiagen). One hundred microliters of supernatant and 100 µl of 100% ethanol were added to a DNA spin column and DNA was recovered following standard protocol (starting at step 5) by Qiagen Stool Kit (Qiagen). DNA was eluted with 30 µl of water and samples were diluted to a final concentration of 20 ng µl. DNA isolated as described above was sequenced using Illumina sequencing technology. DNA libraries were prepared with various insert sized and sequenced using the Illumina GAII sequencing system. In total, 37 Gbases, 8.7 Gbases, 7.5 Gbases, 47.1 Gbases of raw sequence data was produced from 500 bp, 2 kb, 5 kb and 10 kb insert DNA libraries, respectively.

**Figure 2:**
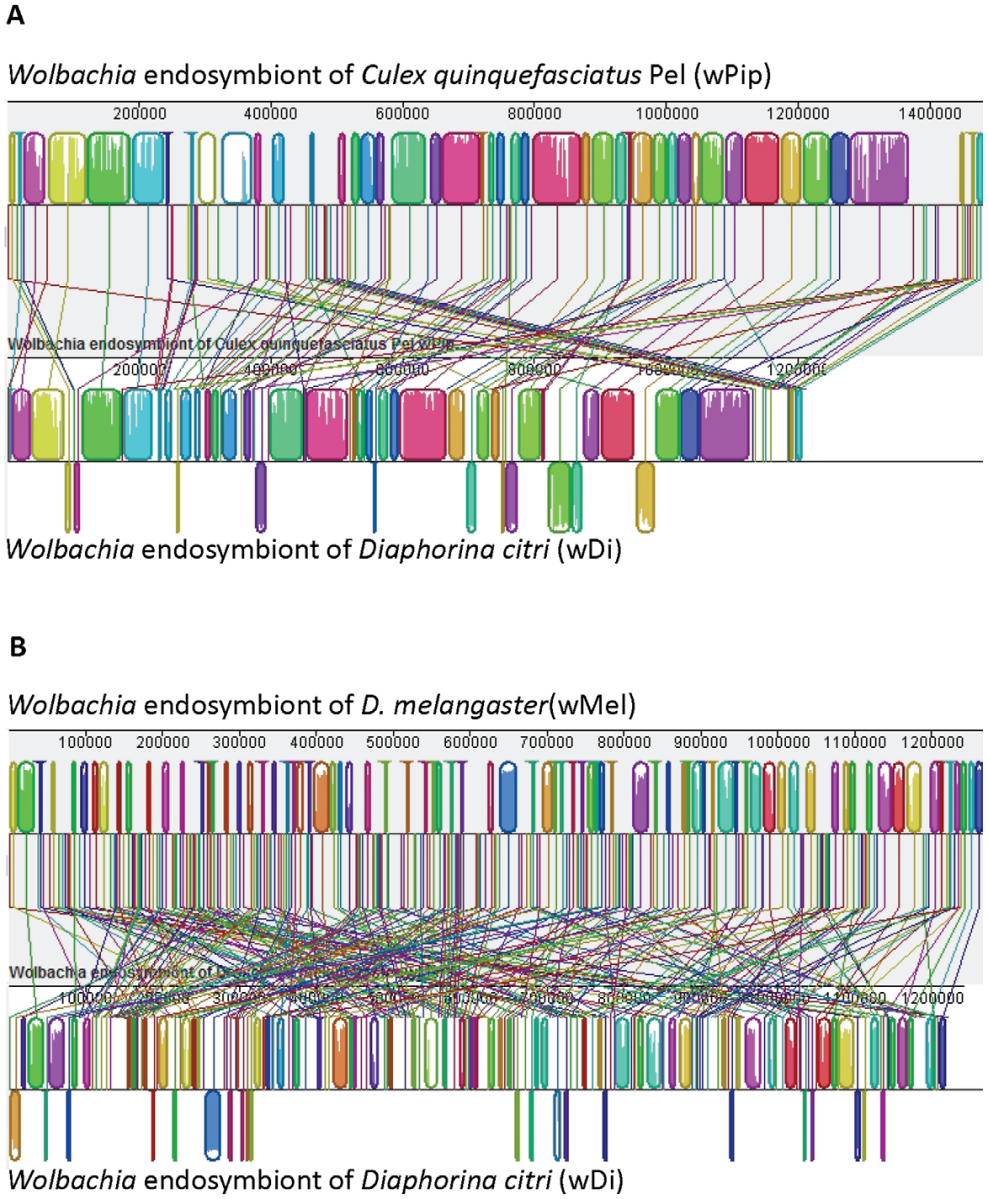
MAUVE alignment of *Wolbachia* endosymbiont of *Diaphorina citri* (wDi) contigs with complete Wolbachia genomes. (A) Alignment with *Wolbachia endosymbiont of Culex quinquefasciatus Pel* (wPip) and (B) alignment with *Wolbachia* endosymbiont of *D. melangaster* (wMel).

**Table 2:** Comparison of genome properties for *Wolbachia* endosymbiont of *Diaphorina citri* (wDi) with the complete genome sequences for four Wolbachia strains^a^.

Wolbachia strain	wDi	wPip	wRi	wBm	wMel
protein coding genes	1213	1275	1150	805	1195
structural RNAs	36	37	39	37	39
size	1.21	1.48	1.45	1.08	1.27
GC %	34	34	35	34	35
% coding	84	81	77	66	80

a Wolbachia strains as follows: wPip (NC_010981), wRi (NC_012416), wBm(NC_006833), wMel (NC_002978).

### Alignment and Mining of Next Generation Sequence Reads for Endosymbiont Characterization

Short read sequence technologies are increasingly being used to generate data from diverse metagenomes, presenting the growing challenge of how best to sort metagenome data into its component organisms. Assemblies are typically generated from short reads and taxonomically classified by comparison with all sequences deposited at Genbank, biasing output to those organisms with high coverage, and frequently resulting in discarding of low coverage or unassembled reads. In the workflow employed here (diagrammed in Figure 1), quality trimmed reads from the *D. citri* metagenome were mapped to reference genomes representing candidate endosymbionts using bowtie2 NGS read aligner [17] in local alignment mode with a word length of 20 bases and allowing 2 mismatches within the word. For these analyses Illumina reads were considered to be probes and the regions of reference genome hit to be “illuminated”. BEDtools [18] and SAMtools [19] were used to determine coverage of aligned ACP metagenome reads over the endosymbiont reference genome and illuminated regions (minimum length 300 bp) were retrieved for further analysis. Neighboring illuminated regions on a reference genome were consolidated if the distance was less than 5% of the combined length of the adjacent illuminated regions in question. Ribosomal regions were screened out from illuminated regions to prevent coverage artifacts due to partial hits from reads to highly conserved portions of the genome. Specificity was evaluated by Blastn analysis of illuminated regions against Genbank nr and the most specific taxonomic assignment determined according to the identity cutoff used.

**Table 3:** Identification of core genome for Wolbachia strains with complete genome sequences using OrthoMCL^a^.

Wolbachia strain	Total proteins	Total core	% core	Total shared	% shared	Total lineage specific	% lineage Specific
wMel	1195	675	56.5	304	25.4	216	18.1
wPip	1275	674	52.9	320	25.1	281	22.0
wRi	1150	672	58.4	407	35.4	71	6.2
wBm	805	670	83.2	54	6.7	81	10.1

a Wolbachia strains as follows: wPip (NC_010981), wRi (NC_012416), wBm(NC_006833), wMel (NC_002978).

### Assembly and Scaffolding of *Wolbachia* Genome

Paired-end and mate-pair Illumina datasets for the psyllid (ACP) metagenome were quality screened at Q2. Reads for the *Wolbachia* endosymbiont of *Diaphorina citri* (strain wDi) were filtered from the *D. citri* metagenome using the complete genome sequences for strains wMel, wBm, wPip, and wRi. Each read pair was blasted against a database of the four sequenced *Wolbachia* genomes. All read pairs that had a 90–100% match and expected insert size were selected for the next step.

The short insert Illumina paired-end reads had coverage of 106X while the large insert mate-pair reads had 4X coverage of the wACP genome. The wACP genome was estimated to be 1.32 Mb. The putative wACP reads were then assembled using Velvet [20] and MIRA3 [21] assemblers over a range of parameter settings. Minimus2 [22] and SSPACE [23] were iteratively used to find overlaps and bridge gaps among the contigs and the wACP scaffolds further improved using Abacas [24] and Mauve contig mover [25] with wPip as reference genome to orient and order the contigs.

**Figure 3:**
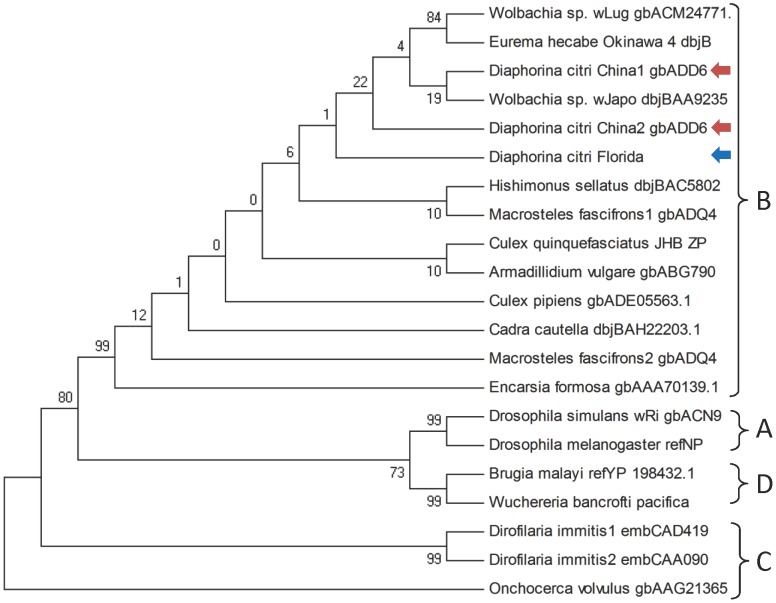
Maximum Parsimony tree for *Wolbachia* FtsZ sequences supporting placement of *Wolbachia* endosymbiont of *Diaphorina citri* (wDi) in supergroup B. Chinese strains are indicated with red arrows and the Florida strain with a blue arrow.

**Figure 4:**
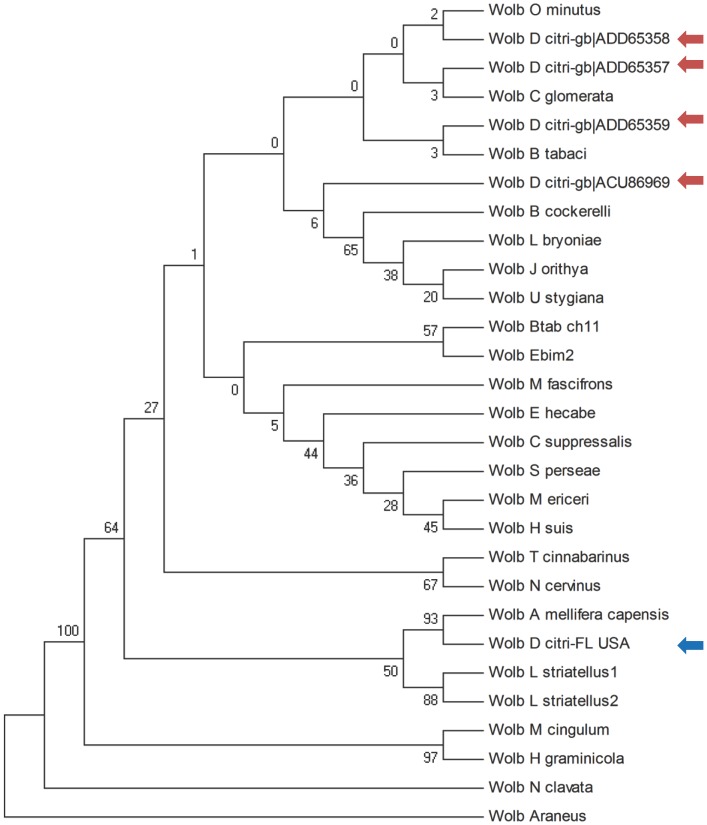
Maximum Parsimony tree for *Wolbachia* Wsp sequences highlighting the placement of Florida (blue arrow) and Chinese isolates (red arrows) in different sub-clades of supergroup B.

**Table 4:** Wolbachia wsp gene sequence accession numbers used in phylogenetic analysis.

Species	Accession number
Wolb._Diaphorina_citri _FL_USA	this publication
Wolb_Apis_mellifera capensis	gb AAN03843.1
Wolb_Laodelphax_striatellus1	gb ACI49289.1
Wolb_Cotesia_glomerata	dbj BAC22086.1
Wolb_D_citri	gb ACU86969.1 China:Beihai
Wolb_D_citri	gb ADD65357.1 China:LiuZhou
Wolb_D_citri	gb ADD65358.1 China:FuZhou
Wolb_D_citri	gb ADD65359.1 China:Shenzhen
Wolb_Orius_minutus	dbj BAC22146.1 (Kumamoto)
Wolb_Chilo_suppressalis	gb AEA30009.1
Wolb_Tetranychus_cinnabarinus	gb AAY41893.1
Wolb_Junonia_orithya	dbj BAC22177.1
Wolb_Ussuriana_stygiana	dbj BAC22178.1
Wolb_Metaphycus_ericeri	gb ADN52161.1
Wolb_Scirtothrips_perseae	gb AAY86157.1
Wolb_Bemisia_tabaci	gb ADD10625.1
Wolb_Eurema_hecabe	dbj BAC22188.1 Okinawa 4
Wolb_Haematopinus_suis	gb AAT08985.1
Wolb_Encarsia_bimaculata	gb ADD10626.1
Wolb_Macrocentrus_cingulum	gb ACZ37424.1
Wolb_Hylyphantes_graminicola	gb ACE00529.1
Wolb_Naupactus_cervinus	gb ACV40686.1
Wolb_Nephila_clavata	gb ABR13649.1
Wolb_Araneus_sp.	gb AAS57559.1
Wolb_Bemisia_tabaci	gb AAN77831.1
Wolb_Macrosteles_fascifrons	gb ADQ43479.1
Wolb_Laodelphax_striatellus2	gb ADC29554.1
Wolb_Liriomyza_bryoniae	dbj BAE79163.1
Wolb_Bactericera_cockerelli	gb AEM06068.1

The resulting wACP contigs from different assemblies were annotated using the RAST [26] pipeline, manually reviewed and edited, and the predictions compared to annotations of *Wolbachia* endosymbiont of *Culex quinquefasciatus* (wPip), the closest sequenced relative of wACP. Sequence data has been deposited at NCBI under Bioproject PRJNA29451, The annotated pseudomolecule for wDi (text S5) can be downloaded and visualized using Artemis [27] or viewed directly in the GBrowse genome viewer at http://citrusgreening.org/.

### Protein Homology and Phylogeny

All 1217 predicted wACP proteins were compared against proteins from the four sequenced *Wolbachia* genomes using OrthoMCL [28]. Core proteins are present in all members of a clade while shared proteins are proteins that are present in one or more members. Protein sequence similarity was determined by NCBI Blast [29] at a range of E values and percent identity cutoffs with varying stringency settings compared. Core and shared assignments were made when the classification was highly conserved (>80%) across all runs. To determine the representation of the core *Wolbachia* proteins in the wACP scaffold, all wACP proteins were Blasted against a database of labeled *Wolbachia* proteins. 1164/1213 wACP proteins had hits of which 670 were to core proteins.

Alignments of FtsZ and Wsp sequences were generated using ClustalW2 [30] and Maximum Parsimony trees constructed using PAUP 4.0b2 [31] and MEGA4 [32].

## Results and Discussion

### Characterization of Bacterial Endosymbionts Represented in the *D. citri* Metagenome Sequence

The goal of the strategy employed here was to maximize detection of endosymbionts in the unassembled metagenome by mapping reads to reference sequences for organisms previously identified by rDNA amplification. In addition to establishing the level of support present in the metagenome sequence for these candidates, this approach further reveals whether coverage levels of any component endosymbionts are sufficient to proceed with draft genome assembly. A total of 17 genomes were selected for endosymbiont identification based on the list of endosymbionts previously identified by rDNA amplification from Florida and Indonesian *D. citri* isolates, with endosymbionts of *B. cockerelli*, the psyllid vector of another *Ca*. Liberibacter pathogen also included (Table 1).

#### Wolbachia

As shown in Table 1, the template genome exhibiting the highest level of read coverage was that of *Wolbachia* endosymbiont of *Culex quinquefasciatus* Pel, with illuminated regions covering over 1.2 Mb or 82% of the reference genome. The titer of *Wolbachia* strains can vary significantly among insects and isolates [33], [34] and the relatively high incidence of reads from the *D. citri* metagenome mapping to the reference *Wolbachia* sequences may reflect a relatively high titer in these samples.

#### *Ca*. Carsonella

In contrast to *Wolbachia*, a relatively low number of reads mapped to the *Ca*. Carsonella ruddii PV reference genome sequence, producing illuminated regions totaling 5 kb or 3% of the genome (Text S1). *Ca*. Carsonella is assumed to be present and the reads accounting for the illuminated regions appear specific for *Ca*. Carsonella as no non-Carsonella nucleotide sequences in Genbank share over 80% identity with these regions. The most likely reason for the low coverage is the previously demonstrated bias of next generation sequencing technologies for regions of DNA with higher GC content [35]. *Ca*. Carsonella strains have the lowest GC content (14–17%) among sequenced bacterial genomes and successful sequencing by Illumina technology has required alternations to standard protocols [7]. Consistent with this explanation, the small number of regions that were illuminated in the reference genome have higher GC content (24%) than the genome overall.

#### Enteric endosymbiont

In contrast to *Wolbachia* and *Ca*. Carsonella, which have been found in psyllid isolates from diverse sources, the repertoire of other psyllid-associated bacteria identified by rDNA amplification vary depending on psyllid species and geographical origin [11], [12]. To identify those candidates supported by the metagenome sequence from the Florida isolate, sequence reads were mapped to reference genome sequences of bacteria identified from multiple *D. citri* isolates and *B. cockerelli*. Among the endosymbionts identified from the Florida *D. citri* isolate by rDNA sequencing was an enteric bacteria closely related to *Klebsiella variicola* and *Salmonella enteric*a [12]. Our read mapping supports the presence of an enteric bacterium, with 604 kb and 387 kb cumulatively illuminated in the genomes of *Salmonella* and *Klebsiella*, equivalent to 12.6% and 7.1% of their respective genomes (Text S2, S3). While ribosomal DNA sequencing was insufficient to distinguish between *Salmonella* and *Klebsiella*, the higher coverage for *Salmonella* shown here suggests that the enteric bacterium represented in the metagenome is more closely related to *Salmonella* than *Klebsiella*. This is further supported by taxonomic analysis of the illuminated regions. 27% of the illuminated regions in *Salmonella* are specific to that genus with the remainder mapping to regions of the *Salmonella* genome that are more generally conserved among enteric bacteria. In contrast, only 14% of illuminated regions in *Klebsiella* are specific to that genus with the remainder being shared with *Salmonella*. Interestingly, while enteric bacteria have been found in the gut microflora of a variety of insects [36]–[38], *Salmonella* is less commonly found than other genera such as *Klebsiella* and *Enterobacter*.

#### Endosymbiont candidates with low coverage

Of the remaining bacteria identified by ribosomal DNA amplification, only *Acidovorax* displayed read coverage exceeding 1% of the genome (Text S4). Taxonomic analysis of the 3% of the *Acidovorax* genome illuminated during read mapping indicated that a quarter of the regions illuminated were specific to *Acidovorax* at the sequence identity cutoff used, with 85% being more generally conserved among the Comamonadaceae. Members of the Comamonadaceae have been found in association with diverse insects [36], [39] and the closely related genus *Verminephrobacter* is known to be a symbiont of earthworms [40].

In contrast, mapping to reference genome sequences for *Acinetobacter*, *Janthinobacterium*, and *Herbaspirillum* yielded illuminated regions of just a few kilobases amounting to less than 1% of these genomes. rDNA amplification from both *D. citri* and *B. cockerelli* revealed a sequence having 99% sequence identity to a *Staphylococcus* isolate, suggesting that bacteria in this genus may be widely distributed among psyllids. However, mapping of the *D. citri* metagenome reads against four different *Staphylococcus* species did not yield any illuminated regions at the cutoffs used. *Methylibium*, *Ralstonia*, and *Bradyrhizobium* have also been reported present in the potato psyllid, *B. cockerelli*, but read mapping did not yield illuminated regions exceeding 1% of the genome. Closer examination of the few kilobases that are illuminated in these cases of exceptionally low coverage indicate that they correspond either to mobile elements such as insertion sequences that are not specific to the genus in question (as in the case of *Herbaspirillium*) or map to regions more broadly conserved across higher taxonomic levels. For example, regions illuminated in *Ralstonia* and *Methylium* are broadly conserved among the Burkholderiales and Comamonadaceae, respectively, corresponding to a subset of the regions illuminated in the *Acidovorax* genome. While the limited coverage observed for these bacteria does not rule out their presence as shown by rDNA sequencing, these data suggest that the major impact on the biology of the *D. citri* and *Ca*. L asiaticus likely derives from *Wolbachia*, *Ca*. Carsonella, and the enteric bacterium.

### Draft Genome Sequence of the *D. citri Wolbachia* Strain

*Wolbachia* are maternally inherited, intracellular, *Rickettsia*-like bacteria known to infect a wide range of arthropods. Recent surveys indicate that as much as 66% of all insect species may be infected with *Wolbachia,* making it one of the most ubiquitous endosymbionts described to date [8]. Infections with this agent have been associated with various reproductive abnormalities in the host, including cytoplasmic incompatibility (CI), the most common phenotype in arthropods, whereby the offspring of uninfected females and infected males fail to develop. CI additionally leads to parthenogenesis in wasps, in which infected virgin females produce infected female offspring, and feminization of genetic males in an isopod species [41]–[43]. The ability of *Wolbachia* to modify the reproductive success of its host enables it to increase in frequency in host populations without the need for horizontal transmission. Introduction of life-shortening *Wolbachia* strains into mosquitoes has proven an effective strategy for control of the vectored virus causing dengue fever [44], [45].

Read mapping to the wPip genome sequence suggested that coverage for *Wolbachia* in the metagenome data was of a level sufficient for generation of a draft genome sequence. To more comprehensively isolate *Wolbachia*-derived reads, the *D. citri* metagenome sequence data was filtered using the complete genome sequences for *Wolbachia* strains wMel, wBm, wPip, and wRi. The resulting read set was assembled and the 167 contigs evaluated for overlaps reducing the total scaffold number to 104. The wDi contigs were aligned with closed Wolbachia genome sequences using MAUVE [25] to gain a better picture of gene conservation and synteny. As shown in Figure 2 and Figure S1, wDi contigs exhibited a higher degree of gene synteny with wPip sequence than with wMel or other Wolbachia genome sequences, resulting in selection of wPip as the reference genome for contig ordering. As shown in Table 2, the number of protein coding genes in wDi is very similar to wPip, though the total genome size is somewhat lower, likely owing to the fact that repeat regions are under-represented in assemblies from short-read sequence data.

To assess completeness of the wDi draft genome, annotated genes in *Wolbachia* strains wPip, wRi, wMel, and wBm were categorized using OrthoMCL (Table 3). A total of 670 core gene clusters were identified for the four genomes using an e value of 10^−5^. Each of the 670 core clusters is represented in the wDi draft genome annotation, with the exception of a single core group composed entirely of hypothetical genes. Small differences in the numbers of genes assigned to core clusters result from instances where gene products were assigned to more than one cluster.

Genes determined by OrthoMCL to be lineage specific in wPip and wDi were manually curated and those arising from different annotation calls in conserved regions eliminated. Blastp analysis of the remaining 32 lineage specific gene products in wDi and 65 lineage specific gene products in wPip was conducted. All of the unique gene products in wDi were of unknown function, with 11 having homologs in strains wAlbB and wAna which, like wPip, are endosymbionts of mosquito [46], [47]. Of the 65 gene products present in wPip but absent from wDi, 40 are hypothetical and 16 correspond to mobile elements. Those with known function include a predicted glyoxylase and an aminoglycoside phospho-transferase, both associated with antibiotic resistance.

#### Ankyrin domain proteins

Among the most interesting proteins encoded by Wolbachia strains are those having ankyrin domains, characterized by the presence of tandemly arranged 33-residue long repeats of variable number but sufficiently divergent at the nucleotide level to permit assembly even when sequenced by short read technologies. Typically associated with eukaryotes, ankyrin proteins have been shown to mediate protein-protein interactions [48]. They are secreted by other members of the Anaplasmataceae and interact with host DNA and/or protein [49], [50]; it has been speculated that reproductive manipulation of host by *Wolbachia* might be achieved through ankyrin binding of host proteins [51], [52].

The number of ankyrin proteins varies among sequenced *Wolbachia* strains, with as few as five in wBm to as many as 60 in wPip [53], [54]. Annotation of the wDi genome revealed the presence of 54 predicted proteins containing ankyrin repeats (Text S6). Blastp analysis of these against the four closed Wolbachia genomes reveals that four of the predicted wDi ankyrin gene products are common to all of these genomes. Of the remaining 50, 38 exhibit a high level of similarity with those encoded by wPip, 10 and 11 with wMel and wRi, respectively, and two with wBm. Twenty-five of those shared with wPip are also present in the three draft sequences for other mosquito-associated Wolbachia strains from *Culex quinquefasciatus* (JHB [55], wAlbB [47]), and from *C. pipiens molestus*, suggesting that the mosquito may be a useful model for understanding psyllid-*Wolbachia* interactions (Table S1).

Extensive studies attempting to correlated ankyrin protein repertoire and/or expression with reproductive impacts such as cytoplasmic incompatibility suggest a complex relationship involving a network of factors [51], [56], [57]. A homolog of the phage-associated pk2 group of ankyrin proteins which correlates with cytoplasmic incompatibility in *Culex* [51] and feminization in isopods [52] is present in one of the two wDi phage regions.

That said, there are also significant differences in the ankyrin repertoire between wDi and mosquito-associated strains. Twelve predicted wDi ankyrin proteins diverge significantly from previously characterized Wolbachia ankyrin proteins. Although five cases of apparent divergence likely result from fragmentation due to contig boundaries, seven predicted ankyrin proteins represent candidates for involvement in a psyllid-specific host-endosymbiont interaction. Conversely, 11 of the ankyrin protein encoding genes in wPip do not have close homologs in wDi, including four ankyrin proteins noteworthy for their length and present in two or more of the other mosquito-associated *Wolbachia* strains: WP0293 (5.9 kb); WP0292 (8.2 kb); WP0407 (7.8 kb); WP0462 (7.9 kb). These four gene products share regions of similarity with one another and with two non-ankyrin proteins conserved in both wDi and the mosquito-associated strains (WP0364 and WP1346 in wPip) indicative of a rapidly evolving family derived in part from the non-ankyrin genes. The presence of WP0364 and WP1346 homologs in wDi suggesting that the wPip and wDi lineages split off prior to the evolution of this family [54].

#### Type IV secretion

Ankyrin proteins produced by *Legionella pneumophila* and *Coxiella burnetii* [58], and by *Anaplasma phagocytophilum* which is in the same family as *Wolbachia* [59], are secreted by the type IV secretion system. This has led to speculation that *Wolbachia* may employ the Type IV secretion system to secrete ankyrin proteins or other effectors involved in manipulation of host biology [60]. A two cluster arrangement of Type IV secretion genes is widely conserved in *Wolbachia* genomes [60], [61], and appears to be shared by wDi. The arrangement of the type IV secretion genes in wDi aligns with the clusters in wPip and with alignment extending into flanking genes; the only exception being the second copy of virB9 copy which in the wDi draft is on a contig of its own, preventing evaluation of flanking genes (Figure S2).

#### Nutritional provisioning

Many insect endosymbionts provide a fitness advantage to their hosts through metabolic provisioning and it has been proposed that a nutritional relationship with the host may enhance selection for *Wolbachia* infection particularly for strains that have successfully invaded host populations in the absence of reproductive manipulation [62]. Kremer et al have demonstrated that *Wolbachia* can alter iron homeostasis in both hosts for which it is an obligate mutualist as well as in cases of facultative parasitism. They speculate that by reducing iron toxicity in cases of high iron, *Wolbachia* may provide a selective advantage to its hosts. [63]. The required bacterioferritin gene is present in all sequenced *Wolbachia* strains including wDi.

In contrast to well-studied insect-endosymbiont systems like that between aphids and *Buchnera*, there is no evidence for *Wolbachia* providing its host with essential nutrients such as amino acids. However, given the extent of gene loss, *Wolbachia* is clearly nutritionally dependent upon its host. Predicted metabolic pathways and transporters have been tallied in both the wMel and wBm *Wolbachia* strains revealing retention of pathways for glycolysis, pentose phosphate pathway, purine metabolism and catabolism of select amino acids in addition to transporters for diverse substrates including carbohydrates, amino acids, and inorganic cations [53], [64]. Comparison of the fully sequenced genomes and wDi reveals conservation of these remaining metabolic genes and transporter genes among *Wolbachia* genomes, with variation observed only for three transporters that are limited to the wMel and wRi genomes. *Ca*. Liberibacter is a reduced genome bacterium dependent on its host plant or insect vector for provisioning of many essential nutrients. Comparison of the predicted metabolic capabilities of wDi and *Ca*. L. asiaticus reveals several metabolic capabilities present in wDi and absent from *Ca*. L. asiaticus, including the ability to synthesize thiocysteine, homocysteine, methylmalonyl-CoA and L*-*erythro*-4-*hydroxyglutamate from precursor compounds. However, there is no evidence for an evolved symbiotic relationship involving the provisioning of *Ca*. L. asiaticus with essential nutrients.

#### DNA repeat analysis

Wide variation has been observed among *Wolbachia* strains regarding the proportion of the genome comprised of repeated sequences, with strain wRi having the highest (22.1% of the total genome) and others significantly lower (wMel = 14%; wBm = 5.4%). Draft genome sequences derived from short-read next generation technologies typically underestimate the extent of repeated regions owing to the difficulty of assembling non-unique sequences. However, repeat characterization provides a valuable tool for future development of strain-specific diagnostic markers, and analysis with RepeatMasker [65] and RepeatScout [66] succeeded in identifying known and novel repeats in the wACP scaffold including 16 *ab-initio* repeat families with an average length of 184 bp and comprising 20315 bp or 1.63% of the wACP scaffold. A total of 196 known repeats with an overall length of 9256 bp (0.74%) were identified by RepeatMasker. A majority of known repeats are either small RNA or low complexity regions (Text S7). The annotated draft genome sequence for wDi, including the locations of predicted ankyrin proteins and repeat sequences, can be viewed on the GBrowse genome viewer at http://citrusgreening.org/.

### Phylogenetic Characterization of *Wolbachia*

Genetic differences among populations of *D. citri* and associated endosymbionts hold potentially important insights into differences in vector behavior and their contribution to geographical variations in the spread and control of citrus greening. For instance, several research groups have shown that the parasitoid wasp *Tamarixia radiata,* introduced in the New World to control invasive *D. citri* populations, varies significantly in effectiveness depending on geographical location [67]–[71] and as previously discussed, the complement of endosymbionts in the *D. citri* metagenome appears to vary in relation to isolate origin [11], [12].

Accumulated phylogenetic analyses indicate that the Florida *D. citri* isolates cluster with *D. citri* populations in Southwest Asia, distinct from *D. citri* populations of in China [72]. Supporting data include analyses of the *D. citri* CoxI protein sequence [72], as well as comparison of prophage gene sequences from *D. citri*-derived *Ca.* Liberibacter asiaticus. Sequence variation in the phage terminase gene between Guangdong and Yunnan strains show they are highly similar or identical suggestive of a common recent origin, while the single Florida strain evaluated showed significantly more divergence [73].

To determine whether *Wolbachia* phylogeny supports the same pattern, the FtsZ and Wsp gene products of wDi were analyzed. The sequence of the cell division protein FtsZ is routinely used for placement of *Wolbachia* strains into the established supergroups A–F [74]. Supergroups A and B include *Wolbachia* spp. from arthropods only, while known members of supergroups C and D are restricted to filarial nematodes. *Wolbachia* spp. from the Collembolan *F. candida* represent a divergent lineage, named supergroup E by [75] and supergroup F comprises representatives of filarial nematodes (*Mansonella* spp.) and the termite *Kalotermes flavicollis* [76]–[78]. Phylogenetic analysis of the FtsZ sequences from *Wolbachia* in diverse *D. citri* isolates clearly places wDi within *Wolbachia* supergroup B, confirming the previously observed superior alignment of the wDi draft genome to supergroup B strain wPip (Figure 3). The FtsZ phylogenetic tree also supports the hypothesis that *Wolbachia* strains from the Chinese *D. citri* isolates fall within a different clade than the Florida isolate characterized here. Distinction between Chinese isolates and the Florida isolate is further supported by phylogenetic analysis of Wsp, an outer membrane protein frequently used for distinguishing relationships among more closely related strains [79] (Figure 4, Table 4). Interestingly, the *Wolbachia* strain present in *B. cockerelli*, the psyllid vector of *Ca*. Liberibacter solanacearum clusters with the four Chinese wDi isolates.

Sequence diversity in *D. citri*, wDi, and Las underlies variation in the biology of citrus greening disease, including but not limited to observed differences in parasatoid effectiveness. In combination with the availability of primary cell cultures for *D. citri*-USA [80], genome sequence data for the Florida isolate of *D. citri* (http://www.sohomoptera.org/), for a Florida isolate of *Ca*. L. asiaticus [81], and for the *Wolbachia* endosymbiont described here provides a valuable basis for comparison from which to explore the genetic sources of variation in vector and disease biology for citrus greening disease worldwide.

### Conclusions

1. Read mapping of the *D. citri* metagenome sequences to reference genomes supports the presence of *Wolbachia*, an enteric bacterium most similar to *Salmonella*, and more limited support for a member of the Comamonadaceae.
2. *Wolbachia*-derived reads were extracted using the complete genome sequences for four *Wolbachia* strains and a draft genome for wDi was assembled.
3. Genome alignment indicates membership of *Wolbachia* wDi in supergroup B, further supported by phylogenetic analysis of FtsZ. FtsZ and Wsp phylogenies additionally indicate that the *Wolbachia* strain in the Florida *D. citri* isolate falls into a sub-clade of supergroup B, distinct from *Wolbachia* present in Chinese *D. citri* isolates.
4. Candidate host interaction factors encoded by the wDi genome include 54 ankyrin repeat-containing proteins, a Type IV secretion pathway, and a bacterioferritin gene linked to iron homeostasis in the host. Several metabolic capabilities were identified in wDi that are absent from *Ca*. L. asiaticus, the causal agent of citrus greening that is transmitted by *D. citri*.

## Supporting Information

Figure S1 **MAUVE alignment of** ***Wolbachia*** **endosymbiont of** ***Diaphorina citri*** **(wDi) contigs with the genomes of the four fully sequenced** ***Wolbachia*** **strains: (A)** ***Wolbachia*** **endosymbiont of** ***Brugia malayi*** **(wBm), (B)** ***Wolbachia*** **endosymbiont of** ***D. simulans*** **(wRi), (C)** ***Wolbachia*** **endosymbiont of** ***D. melangaster*** **(wMel), and (D)** ***Wolbachia*** **endosymbiont of** ***Culex quinquefasciatus Pel*** **(wPip).**
(PPTX)

Figure S2 **Alignment of** ***Wolbachia*** **endosymbiont of** ***Diaphorina citri*** **(wDi) and** ***Wolbachia*** **endosymbiont of** ***Culex quinquefasciatus Pel*** **(wPip) genes encoding the Type IV secretion system.**
(PPTX)

Table S1 **Summary of predicted ankyrin proteins in the wDi draft genome highlighting coordinates, sequence and representation in the closed Wolbachia genomes wRi, wBm, wMel, and wPip, and the draft genome sequences of Wolbachia from mosquito associated strains JHB (PRJNA32209), wAlbB (PRJNA81759), and wPip Mol (PRJEA52451).**
(XLSX)

Text S1 **Text S1. Coordinates of regions in the** ***Candidatus*** **Carsonella ruddii PV genome illuminated by** ***D. citri*** **metagenome sequences.** Regions can be visualized by loading the Genbank accession for the sequence (AP00918) and this file into the Artemis Genome Viewer (http://www.sanger.ac.uk/resources/software/artemis/).
(GFF)

Text S2 **Text S2. Coordinates of regions in the** ***Salmonella enterica*** **subsp.** ***enterica*** **Typhi Ty2 genome illuminated by** ***D. citri*** **metagenome sequences.** Regions can be visualized by loading the Genbank accession for the sequence (AE014613) and this file into the Artemis Genome Viewer (http://www.sanger.ac.uk/resources/software/artemis/).
(GFF)

Text S3 **Coordinates of regions in the** ***Klebsiella variicola*** **genome illuminated by** ***D. citri*** **metagenome sequences.** Regions can be visualized by loading the Genbank accession for the sequence (CP001891) and this file into the Artemis Genome Viewer (http://www.sanger.ac.uk/resources/software/artemis/).
(GFF)

Text S4 **Coordinates of regions in the** ***Acidovorax avenae*** **subsp.** ***avenae*** **genome (NC_015138) illuminated by D. citri metagenome sequences.** Regions can be visualized by loading the Genbank accession for the sequence (CP002521) and this file into the Artemis Genome Viewer (http://www.sanger.ac.uk/resources/software/artemis/).
(GFF)

Text S5 **Annotated pseudomolecule of the wDi draft genome sequence in Genbank format created by concatenation of contigs aligned to the wPip genome sequence.** Contigs are separated by the linker sequence nnnnnttaattaattaannnnn.
(GBK)

Text S6 **Predicted wDi ankyrin proteins in Genbank format**. Proteins can be visualized by loading the wDi pseudomolecule and this file into the Artemis Genome Viewer (http://www.sanger.ac.uk/resources/software/artemis/).
(GBK)

Text S7 (GFF)
