# Supplementary figures and images for: Survey of Endosymbionts in the Diaphorina citri Metagenome and Assembly of a Wolbachia wDi Draft Genome
Source: PLoS One. 2012 Nov 16;7(11):e50067. doi: 10.1371/journal.pone.0050067 (PMC3500351; doi:10.1371/journal.pone.0050067)

## Slide 1
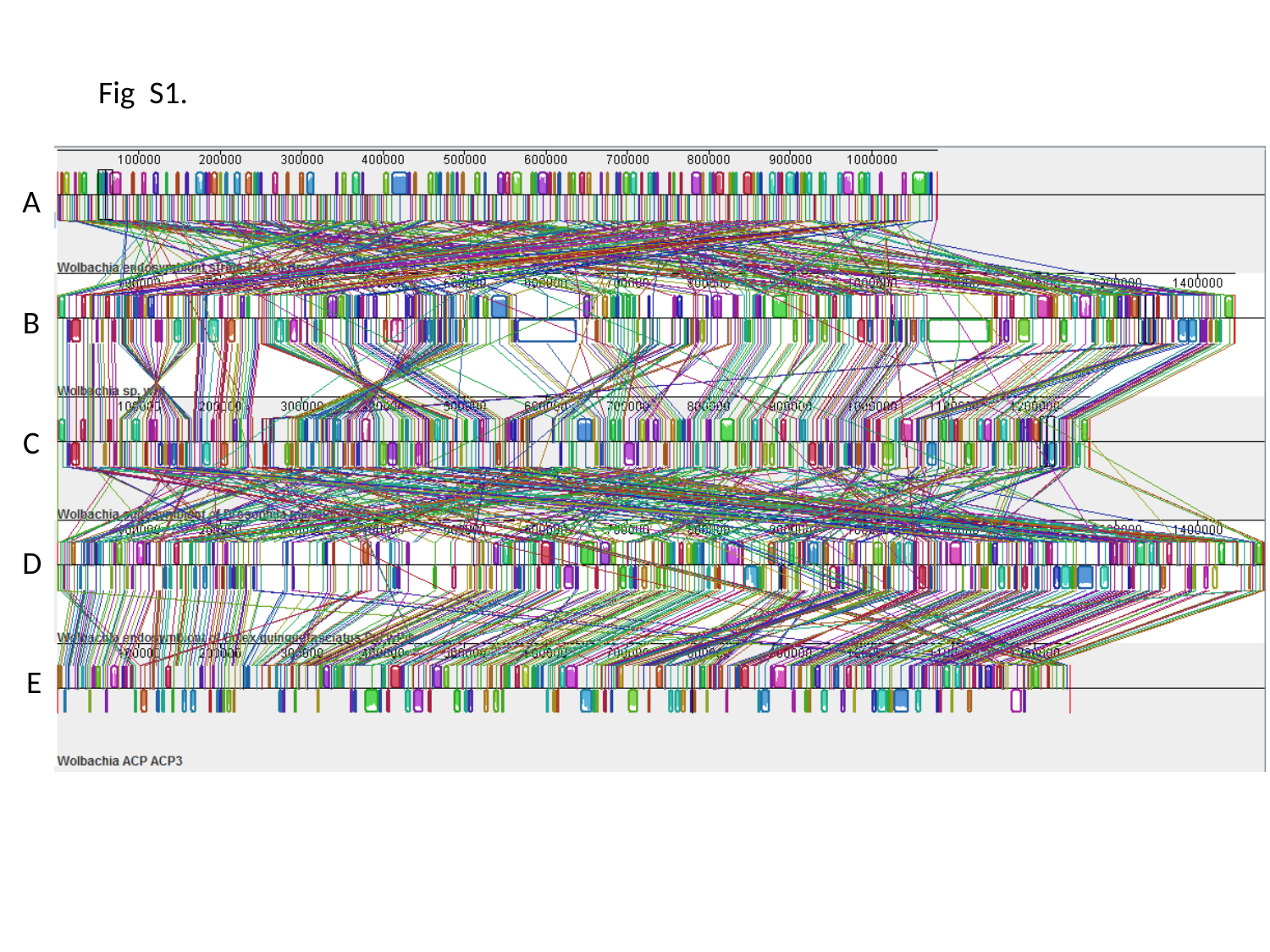

Fig S1.
A
B
C
D
E

Supplement: Figure S1 — MAUVE alignment of Wolbachia endosymbiont of Diaphorina citri (wDi) contigs with the genomes of the four fully sequenced Wolbachia strains: (A) Wolbachia endosymbiont of Brugia malayi (wBm), (B) Wolbachia endosymbiont of D. simulans (wRi), (C) Wolbachia endosymbiont of D. melangaster (wMel), and (D) Wolbachia endosymbiont of Culex quinquefasciatus Pel (wPip). (PPTX) [file pone.0050067.s001.pptx]

## Slide 1
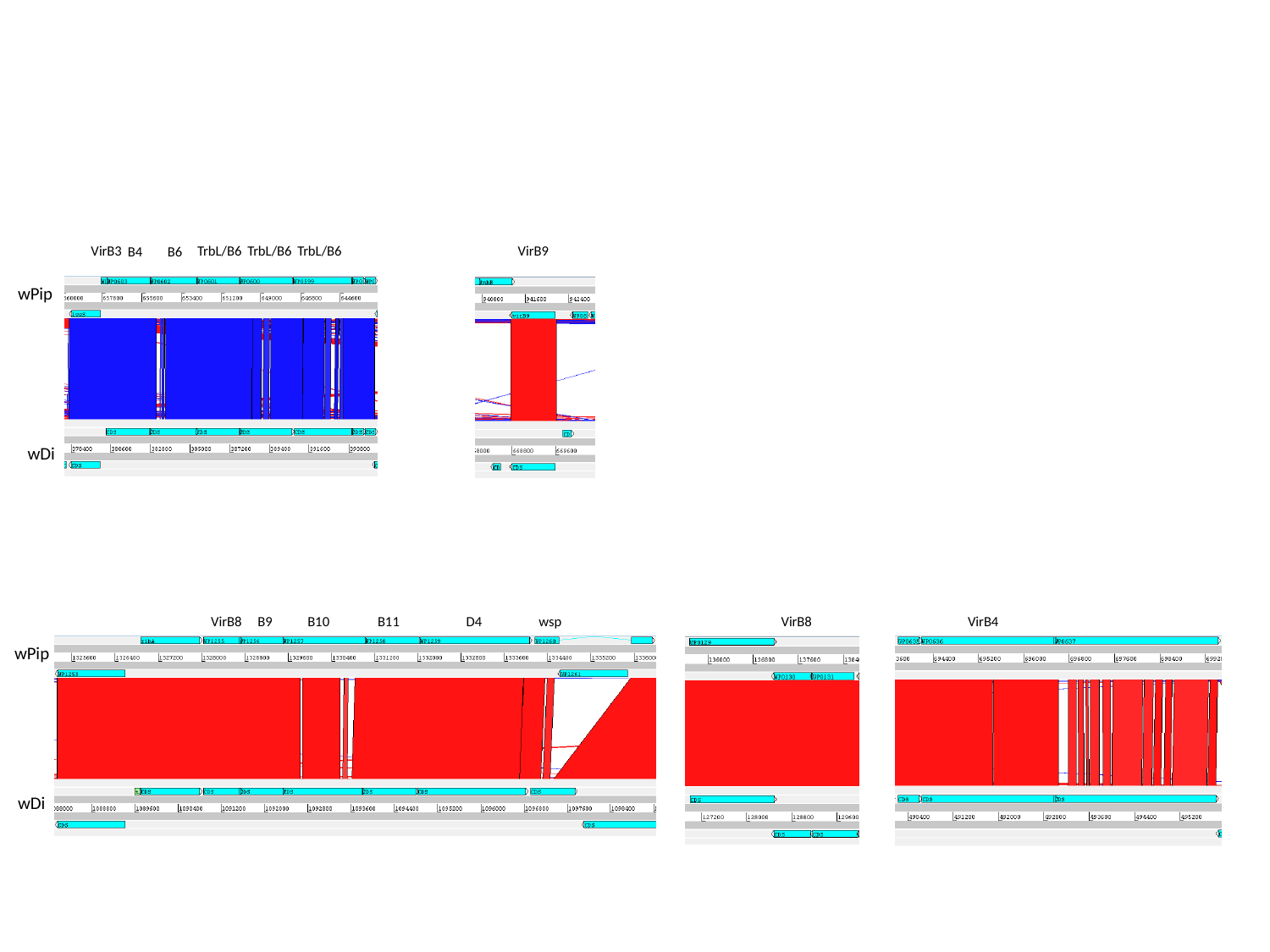

VirB3
TrbL/B6
TrbL/B6
TrbL/B6
VirB9
B4
B6
wPip
wDi
VirB8
B9
B10
B11
D4
wsp
VirB8
VirB4
wPip
wDi

Supplement: Figure S2 — Alignment of Wolbachia endosymbiont of Diaphorina citri (wDi) and Wolbachia endosymbiont of Culex quinquefasciatus Pel (wPip) genes encoding the Type IV secretion system. (PPTX) [file pone.0050067.s002.pptx]
